# Lack of age-related mosaic loss of W chromosome in long-lived birds

**DOI:** 10.1098/rsbl.2021.0553

**Published:** 2022-02-23

**Authors:** Nancy Trujillo, Mónica Martínez-Pacheco, Cecilia Soldatini, Sergio Ancona, Rebecca C. Young, Yuri V. Albores-Barajas, Alberto H. Orta, Cristina Rodríguez, Tamas Székely, Hugh Drummond, Araxi O. Urrutia, Diego Cortez

**Affiliations:** ^1^ Centro de Ciencias Genómicas, UNAM, CP62210, Cuernavaca, México; ^2^ Laboratorio de Biología Celular y Molecular, Facultad de Ciencias Naturales, Universidad Autónoma de Querétaro, CP76010, Querétaro, México; ^3^ Centro de Investigación Científica y Educación Superior de Ensenada - Unidad La Paz, Calle Miraflores 334, CP23050, La Paz, Baja California Sur, México; ^4^ Instituto de Ecología, UNAM, Ciudad Universitaria, CP04510, Ciudad de México, México; ^5^ CONACYT. Consejo Nacional de Ciencia y Tecnología, Av. Insurgentes Sur 1582, Col. Crédito Constructor. Alcaldía Benito Juárez, CP03940, Ciudad de México, México; ^6^ Universidad Autónoma de Baja California Sur., Km. 5.5 Carr. 1. La Paz, Baja California Sur, México; ^7^ Milner Centre for Evolution, Department of Biology and Biochemistry, University of Bath, Bath BA2 7AY, UK; ^8^ Department of Evolutionary Zoology and Human Biology, University of Debrecen, Debrecen H-4032, Hungary

**Keywords:** mosaic loss of chromosome W, *Sula nebouxii*, *Fregata magnificens*, ageing

## Abstract

Females and males often exhibit different survival in nature, and it has been hypothesized that sex chromosomes may play a role in driving differential survival rates. For instance, the Y chromosome in mammals and the W chromosome in birds are often degenerated, with reduced numbers of genes, and loss of the Y chromosome in old men is associated with shorter life expectancy. However, mosaic loss of sex chromosomes has not been investigated in any non-human species. Here, we tested whether mosaic loss of the W chromosome (LOW) occurs with ageing in wild birds as a natural consequence of cellular senescence. Using loci-specific PCR and a target sequencing approach we estimated LOW in both young and adult individuals of two long-lived bird species and showed that the copy number of W chromosomes remains constant across age groups. Our results suggest that LOW is not a consequence of cellular ageing in birds. We concluded that the inheritance of the W chromosome in birds, unlike the Y chromosome in mammals, is more stable.

## 1. Introduction

Many sex chromosomes in amniote species originated greater than 50 Myr [1–5] following the emergence of genes that acted as regulators of gonadal development [6]. Mammals show X/Y sex chromosomes, whereas birds have Z/W sex chromosomes. During evolution, Y and W chromosomes underwent recombination arrests to preserve the sex-determining loci, a process that is often associated with the accumulation of repetitive DNA and massive genetic loss due to large-scale deletions [7–9].

Recently, it has been shown that sex-specific survival is more strongly associated with the type of sex chromosome system (X/Y or Z/W) than with typical ecological factors [10]. In general, the sex that carries the sex-limited chromosome (Y/W) dies earlier in vertebrates and invertebrates [11]. In birds, specifically, males live longer than females [12], a pattern likely caused by still unknown genetic factors linked to the W chromosome that may affect female survival. One hypothetical cause is particularly appealing: mosaic loss of the sex-limited chromosome during ageing. It has been noticed that mosaic loss of chromosome Y (LOY) in blood cells of aged men is strongly associated with reduced life expectancy [13–16]. We recently showed that LOY is likely shared across mammals [17], but its presence in species with other sex chromosomes is still unresolved.

It has long been debated whether senescence in birds is analogous to that in mammals [18] because birds do not show clear external signs of ageing. However, both taxa evolved endothermy, and higher body temperatures appear to foster cellular senescence [19–21]. Seabirds are among the birds with the longest longevity [22]; for example, the blue-footed booby (*Sula nebouxii*) can live up to 22 years [23,24], and the magnificent frigatebird (*Fregata magnificens*) up to 30 years [25]. Adult populations of the blue-footed booby are slightly male-biased and those of *F. magnificens* are strongly male-biased (male/female ratios of greater than 1 and greater than 2, respectively; electronic supplementary material, figure S1) [26–28]. We studied a wild population of *S. nebouxii* off the Pacific coast of México that has been monitored over the past three decades and for which we know the exact age of individuals [29,30]. We also analysed data from nestlings and adults of the magnificent Frigatebird from a wild population in Baja California Sur, México. In this work, we tested whether blood cells in long-lived birds evolved age-related mosaic loss of W chromosome (LOW) as a natural consequence of cellular senescence. Based on these sex ratios, we also tested whether LOW could be associated with differential female survival (i.e. we expected higher LOW in *F. magnificens*).

## 2. Material and methods

### (a) Study site and sample collection

Blood samples were used to obtain genomic DNA. Blood samples were obtained in the booby colony of Isla Isabel (21° 52′ N, 105°54′ W), México, where monitoring of birds has been carried out annually since 1989 [29,30]; we sampled 61 females: 13 nestlings–fledglings (0–1 year), 19 young adults (2–7 years), 10 middle-aged adults (8–11 years) and 19 old adults (12–18 years). For the magnificent frigatebird, blood samples were obtained from a population on Isla Espiritu Santo, in Baja California Sur, México. Individuals in this population have been monitored for the past four years; we sampled 41 females: 12 nestlings of 1 month old and 29 adult females of 6–30 years of age (with a likely average of approx. 14 years of age according to the species' population structure [25]). For both species, 0.5 ml of blood was stored in 1 ml of DNA/RNA shield buffer by Zymo Research (cat. no. R1200–125) supplied with 0.3 ml of heparin. Permission for fieldwork and sampling was granted by the Secretaría del Medioambiente y Recursos Naturales (SEMARNAT; permit nos. SGPA/DGVS/08333/10, SGPA/DGVS/05216/20 and SGPA/DGVS/03619/21).

### (b) DNA purification

Purified genomic DNA was required for the analyses and 150 µl of blood was used to purify DNA using the Blood DNA Isolation Mini kit from NORGEN BIOTEK CORP (cat. no. 46300/ 46380).

### (c) RNA purification and sequencing

We generated transcriptomic data to gather genetic information for the blue-footed booby. RNA was purified from blood using the RNAeasy QIAGEN kit. We generated strand-specific RNA-seq libraries, using the Illumina TruSeq Stranded mRNA Library protocol. Each library was sequenced on Illumina HiSeq 2500 platforms at the Macrogene facility in Korea (101 nucleotides, paired-end).

### (d) Assembly of W-linked transcripts in the blue-footed booby

To assemble W-linked sequences in the blue-footed booby we used a subtraction approach that compared male and female transcriptomic data; we used this method previously for other amniote species [1,4,31,32]. Briefly, we removed RNA-seq reads shared between males and females and then used Trinity (v. 2.0.2, k-mer of 25 bp) [33] to assemble a female-specific transcriptome.

### (e) Primer design

The PCR-based method required the design of W, Z and autosomal primers. We worked for the blue-footed booby with the male transcriptome assembly and for the magnificent frigatebird, we worked with a publicly available genome assembly (ASM1338994v1 [34]). We identified genes that could be autosomal or Z-linked by BLASTn [35] searches against orthologous genes on the chicken reference genome (https://www.ensembl.org/Gallus_gallus/Info/Index, v.98). We identified W-linked transcripts from the female-specific transcriptome assembly of the blue-footed booby. We designed primers that amplified around 550 base pairs of exonic sequences using the AmplifX software (v.2.0.7, https://inp.univ-amu.fr/en/amplifx-manage-test-and-design-your-primers-for-pcr). W-specific primers were required to show at least two mismatches with the Z gametologues to increase specificity. For PCR amplification we used the Phusion Flash High Fidelity from Thermo Fisher Scientific (cat. no. F548 L) with male and female genomic DNA. We confirmed the expected copy numbers in males and females using standard qPCR curves. We used four DNA dilutions: 0 ng/µl, 0.2 ng/µl, 2 ng/µl and 20 ng/µl and the PowerUp SYBR Green Master Mix from Thermo Fisher (cat. no. A25741). We chose *NCK2* (autosomal), *VCAN* (Z-linked) and *RICTOR* (W-linked) for the blue-footed booby; and *NCK2* (autosomal), *DMRT1* (Z-linked) and *APC1* (W-linked) for the magnificent frigatebird. We could not use the same Z/W genes in both species due to the lack of the corresponding sequences in the datasets. Primers are provided in electronic supplementary material, table S1.

### (f) Loci-specific PCR and target illumina sequencing

Loci-specific PCR for autosomal, Z and W markers were used as a proxy to quantify the coverage of the sex chromosomes. DNA samples were standardized to 10 ng/µl. We amplified the autosomal, Z-linked and W-linked loci in the same PCR reaction using the Phusion Flash High Fidelity from Thermo Fisher Scientific (cat. no. F548 L). PCR products were purified using Agencourt AMPure XP (cat. no. A63882). PCR products were multiplexed and sequenced in a NextSeq 500 Illumina machine (paired-end, 75 nucleotides long) at UNAM. The quality of the reads was verified using FastQC, and the remaining adaptors were removed with Trimmomatic (v. 036) [36]. Reads were aligned using bowtie2 (v. 2.3.4.1) [37] against the genome sequence of the magnificent frigatebird or the transcriptome assembly of the blue-footed booby. The W-linked gene *APC1* was missing from the genomic sequence of the magnificent frigatebird and was assembled from the sequenced data; the Z gametologue was present in the genomic assembly, which allowed us to confirm the identity of W-specific reads. We then extracted the reads that mapped uniquely to the expected loci and obtained on average 821 199 reads (s.d.: ± 156 573) and 185 578 reads (s.d.: ± 22 995) for the magnificent frigatebird and the blue-footed booby, respectively (electronic supplementary material, table S2). To normalize coverage estimates, we first calculated the difference in coverage for the autosomal marker between individual samples and the median across samples, assuming the same autosomal copy number for all samples of the same species. We then used these values to correct individual W/Z coverages (see electronic supplementary material, table S2 for more details). The median value of nestling birds indicated a copy number of one chromosome. All statistical analyses were performed using the R package, standard libraries. Data were plotted using the R package, ‘ggplot2’ library (https://ggplot2.tidyverse.org).

## 3. Results

### (a) The loci-specific PCR and target sequencing approach

**Figure 1.**
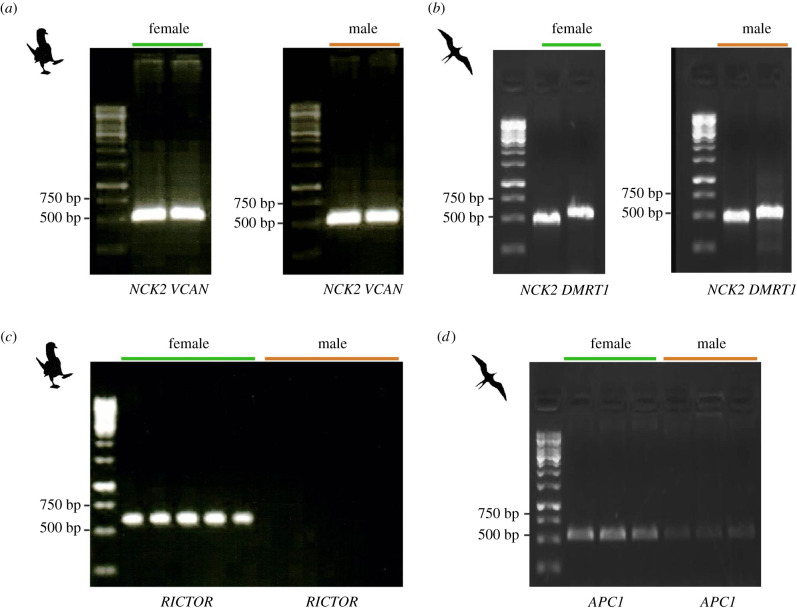
PCR products for the autosomal, Z-linked, and W-linked loci. (*a*) One per cent agarose gel showing a single band of approximately 550 bp for autosomal gene *NCK2* and Z-linked gene *VCAN* in a female and a male of the blue-footed booby. (*b*) Same as (a) for autosomal gene *NCK2* and Z-linked gene *DMRT1* in a female and a male of the magnificent frigatebird. (*c*) The same as in (*a*) but for the W-linked gene *RICTOR* in five females and five males of the blue-footed boobies. (*d*) Same as in (*a*) but for the W-linked gene *APC1* in three females and three males of the magnificent frigatebird; in this case, we observed minor cross-amplification of the Z gametologue in males.

In humans, LOY is generally estimated using data from whole-genomes across age groups. Similar data, however, are lacking for birds. We developed a strategy to estimate LOW using as a proxy the combined amplification and target sequencing of three specific loci (an autosomal, a Z-linked and a W-linked). First, we confirmed that the primers showed the expected pattern: amplification of autosomal and Z loci in both sexes, and amplification of the W locus in females (figure 1).

### (b) The W chromosome is not lost during ageing in seabirds

**Figure 2.**
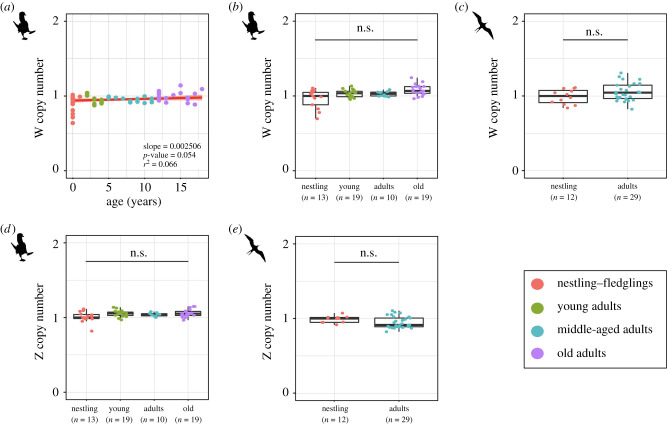
W and Z chromosome copy number estimates across age groups. (*a*) Dot plot of the estimated copy number of W chromosome relative to the age of females in the blue-footed booby. Nestling–fledglings are in red; young adults are in green; middle-aged adults are in blue; old adults are in purple. Significant differences, linear model: lm[W.coverage ∼ age], *p* < 0.05, excluding outliers. (*b*) Box plot of the estimated copy number of W chromosome in the four different age groups of the blue-footed booby: nestlings–fledglings (0–1 year), young adults (2–7 years), middle-aged adults (8–11 years) and old adults (12–18 years). *N*-values are indicated in parenthesis. Significant differences, Benjamin–Hochberg corrected Mann–Whitney U test, *p* < 0.05. (*c*) Box plot of the estimated copy number of W chromosome in the two different age groups of the magnificent frigatebird: nestlings (1 month old) and adults (6–30 years). N values are indicated in parenthesis. Significant differences, Mann–Whitney U test, *p* < 0.05. (*d*) Same as in (*b*) but for the estimated copy number of Z chromosome. (*e*) Same as in (*c*) but for the estimated copy number of Z chromosome.

Sequencing data of Z and W markers were used as a proxy to estimate chromosomal copy numbers across age groups. For the blue-footed booby, we compared the autosomal-normalized coverage of the W-linked locus in 61 females distributed in four different age groups and found no statistically significant differences across age groups (figure 2*a,b*). We also analysed 41 females of the magnificent frigatebird from two different age groups and found that the autosomal-normalized coverage of the W-linked locus was not significantly different between nestlings and adult females (figure 2*c*).

We repeated the analyses using the autosomal-normalized coverage of the Z-specific loci in both species. Again, we did not find statistically significant differences between age groups (figure 2*d–e*).

## 4. Discussion

This is the first evaluation of the occurrence of mosaic loss of the W chromosome in birds. These species have 140 million years old Z/W chromosomes [2] that originated from a different pair of autosomes than the human X/Y system [38], where LOY was reported [15]. We found no signs of LOW during ageing of either species; accumulation of lower coverage values in older individuals, despite technical stochasticity from PCR amplification, would have indicated LOW. Cross-sectional study of wild populations of two long-lived seabirds allowed sampling across a wide age range, particularly in the booby where individuals have been monitored for over three decades, thus, providing the opportunity to explore the genetics of ageing in a bird species. Similar studies can be performed in other birds provided that proper data (DNA samples for individuals of known age and sex across multiple age groups) are available.

In vertebrates and insects, the sex that carries the Y/W chromosome dies earlier [10,11] and because LOY in humans has been correlated with the early death of men [13,15,39], the mosaic loss of sex chromosomes has been proposed as an important process shaping sex-specific survival rates across taxa [40]. Our results, however, are at odds with LOY/LOW reflecting a general process of cellular senescence associated with W chromosomes and/or the evolution of longer lifespans. Our work indicates that LOW does not influence sex-specific survival in seabirds. We could hypothesize that seabirds may be well-buffered against LOW and that alternative genetic or ecological forces are shaping male/female ratios.

We developed a PCR and target sequencing approach to estimate the coverage of W/Z chromosomes using data from an autosomal gene to standardize variations in sequencing depths across samples. This approach allowed us to analyse over 100 samples without the need to sequence whole-genomes. Although further work is needed to establish whether the PCR-based method can detect LOW at low frequencies, our results support the idea that the ploidy of the W chromosome remains constant across age groups, suggesting that the inheritance of this sex chromosome is stable in birds.

Aneuploidies involving the sex chromosomes are among the more frequent chromosomal aberrations in humans [41]. For example, one in 300 newborn babies is aneuploid, most commonly with a missing or additional sex chromosome [42]. By contrast, aneuploidies involving sex chromosomes in birds (ZO karyotype or triploids) are usually lethal at the embryonic stage [43]. Rare cases of adult females with ZZW triploidy have been reported in four species of birds [44–47]. And in chickens, for example, ZZW females develop as inter-sexes [46]. So, it appears that the lack of W chromosomes in birds may be more deleterious than the lack of Y chromosomes in mammals [48,49].

## Data accessibility

RNA-seq data and PCR-based method and target Illumina sequencing data have been deposited to the NCBI-SRA database under BioProject PRJNA764264. Primers used for the PCR-based method can be found in electronic supplementary material, table S1. Raw coverage values and normalized estimates can be found in electronic supplementary material, table S2. Transcriptome for the blue-footed booby, sequence for APC1 (W-linked) used for the magnificent frigatebird, and code used for coverage and statistical analyses is available on Figshare https://figshare.com/articles/dataset/Trujillo_et_al_Lack_of_agerelated_mosaic_loss_of_W_chromosome_in_long-lived_birds/17086718.
